# Mental Health Problems Due to Social Isolation During the COVID-19 Pandemic in a Mexican Population

**DOI:** 10.3389/fpubh.2021.703450

**Published:** 2021-11-23

**Authors:** Alma Delia Genis-Mendoza, José Jaime Martínez-Magaña, María Lilia López-Narváez, Thelma Beatriz González-Castro, Isela Esther Juárez-Rojop, Humberto Nicolini, Carlos Alfonso Tovilla-Zárate, Rosa Giannina Castillo-Avila

**Affiliations:** ^1^Hospital Psiquiátrico Infantil “Juan N. Navarro”, Ciudad de México, Mexico; ^2^Laboratorio de Genómica de Enfermedades Psiquiátricas y Neurodegenerativas, Instituto Nacional de Medicina Genómica, Ciudad de México, Mexico; ^3^Hospital General de Yajalón “Dr. Jesús Manuel Velázquez Siles” Secretaría de Salud, Yajalón, Mexico; ^4^División Académica Multidisciplinaria de Jalpa de Méndez, Universidad Juárez Autónoma de Tabasco, Jalpa de Méndez, Mexico; ^5^División Académica de Ciencias de la Salud, Universidad Juárez Autónoma de Tabasco, Villahermosa, Mexico; ^6^División Académica Multidisciplinaria de Comalcalco, Universidad Juárez Autónoma de Tabasco, Comalcalco, Mexico

**Keywords:** COVID-19, social isolation, mental health, Mexican population, pandemic

## Abstract

**Introduction:** Social isolation due to the COVID-19 pandemic has been identified as a risk factor of several mental disorders. Therefore, the present work aimed to evaluate the effect of social isolation experienced during the COVID-19 pandemic on the mental health of a Mexican population.

**Materials and Methods:** A cross-sectional online survey was conducted in individuals of 18 years of age and over. The questioner was structured to identify onset or worsening of psychiatric symptoms due to social isolation by COVID-19. The survey included changes in eating habits, changes in personal hygiene habits, the starting the use or increased the use of psychoactive substances, symptoms of depression or post-traumatic stress.

**Results:** A total of 1,011 individuals were included in the analysis. The majority were women (68.84%). Changes in eating habits were reported in 38.51% of the participants, 67.80% reported having their physical self-perception distorted or having started a low-calorie diet. Regarding symptoms of depression, 46.10% participants indicated to have at least one depressive symptom, and 4.46% reported suicidal ideation during social isolation. Interestingly, 6.09% of individuals reported that they used to have depressive symptoms prior the COVID-19 pandemic and those symptoms decreased due to social isolation. Additionally, 2.27% of individuals presented symptoms of post-traumatic stress due to the possibility of getting COVID-19.

**Conclusions:** In this work we identified how social isolation has impacted the mental health of the Mexican population. We observed that practically all the symptoms evaluated were affected during isolation, such as personal hygiene and eating habits. Depression and suicidal ideation were the ones that increased the most in the general population, while in individuals who had symptoms of depression before isolation, these symptoms decreased during social isolation.

## Introduction

Human beings are social organisms with complex interrelationships (1). The several ways in which we interact with other humans allow us to form different networks of social interactions which can impact different levels of our environment. One aspect highly impacted by social interaction networks is our health. The study of health implications caused by the modification of social interaction networks is a field that started in the 1970s (2). The main hypotheses are that an increase in social interaction would have health benefits, while social isolation could decrease the individuals' health (3, 4). Social isolation is interpreted as the opposite of the lack of social interaction or integration and is a quantitative measure of the frequency of social contact of an individual (5, 6). In this regard, mental health is possibly the area where social isolation impacts the most. Social isolation has been reported to increase symptoms of depression, suicide attempt and psychosis (4, 7).

In December 2019, a new disease began in China, which the World Health Organization called COVID-19, and in March 2020 it was declared a pandemic (8–10). COVID-19 is caused by a new beta-coronavirus called SARS-CoV2, which affects the lower respiratory tract and manifests as pneumonia (9). The virus is transmitted mainly through respiratory droplets (some >10 μm in diameter), which infected people exhale when talking, coughing, breathing or sneezing. Furthermore, large droplets fall rapidly on surfaces and small droplets can remain suspended in the air, both favoring the spread of the virus (11). For this reason, the governments implemented strategies and measures of social isolation (stay-at-home quarantine orders), visitor restrictions and the closure of public spaces as schools and restaurants to avoid closeness between people and reduce infections (12). Also, the use of face masks was mandatory and cleaning habits such as hand washing, use of antibacterial gel, housekeeping measures and the use of disinfectants inside and outside the home became part of daily life.

However, social isolation to control the dispersion of COVID-19, as well as the change in lifestyle and the constant concern about contracting the virus, could impact the health of people with the appearance of mental problems. In this regard, preliminary studies about the impact of social isolation on mental health, an increase in anxiety and symptoms of depression were reported (13). Nevertheless, the effects of isolation on other aspects of mental health have not yet been explored. On the other hand, it is important to study changes in eating habits such as food throughout the day during social isolation, since depending on the type of diet, they can be problematic and seriously affect your physical and mental health (14). In addition, identifying them in time would allow knowing the behavior of people in social isolation and taking measures to counteract the appearance of eating disorders (serious psychiatric disorders that are characterized by weight control behaviors or abnormal eating) (15). In the same way, the emerging risk of infection by COVID 19 and the constant use of cleaning habits, could affect the mental health of individuals in social isolation and promote the appearance of other mental problems such as obsessive compulsive disorder, but even the moment has not been elucidated.

Therefore, the present work aimed to evaluate the effects of social isolation due to COVID-19 pandemic on the mental health of a Mexican population, through the evaluation of psychiatric symptoms using a survey disseminated on social networks.

## Materials and Methods

### Ethics

Ethical approval was granted by the Research Ethics Committee of the Juárez Autonomous University of Tabasco (103/CIP-DACS/2020) in Mexico. First, we explained the objectives of the study; then the participants signed their consent prior to taking part in this research. Individuals who participated in this study did not receive any financial compensation.

### Participants

This is a cross-sectional online survey study that included a sample of 1,011 Mexican individuals. The online survey was performed from the 30th of March to the 11th of April 2020. The survey was performed during the phase 3 of the COVID-19 pandemic in Mexico, which began on the 21st of March of 2020.

#### Inclusion Criteria

Mexican citizens residing anywhere in the country, of legal age (≥18) who were in social isolation during the COVID-19 pandemic. Only participants without clinical diagnosis or symptoms of COVID-19 and no positive PCR for COVID-19 at the time of the study were included.

#### Exclusion Criteria

Individuals with a positive PCR or an established diagnosis of COVID-19 during or prior to the study, as well as participants with family members diagnosed with the disease.

### Assessment

We designed a questionnaire entitled “Mental Health in the Times of COVID-19,” which was based, adapted and modified from standardized and validated questionnaires (16–21) in accordance with the objectives of our study, and was divided into three sections of interest.

The first section collected sociodemographic data such as gender, age, school level, marital status and occupation. The second and third sections collected behavioral changes in personal hygiene (due to the use of new measures such as constant hand washing during the pandemic), eating habits (to know any change in behavior during the period of social isolation) and the worsening of psychiatric symptoms, due to the fact that compulsory social isolation due to COVID-19 could affect people's mental health and promote the appearance of other mental problems. In addition, for each questions in the last two sections of the questionnaire, participants could select one or more options.

For this study, we defined social isolation (quantitative measure of the frequency of social contact of a person) as the situation of voluntary or mandatory protection of an individual due to the COVID-19 pandemic (5, 6).

#### Cleaning Habits

We include questions to know changes in cleaning habits in the participants. We asked about hand washing habits, which included the number of times a day that participants washed their hands (2 to 5 times a day, 5–8 times a day or 8–16 times a day) or if they did not wash their hands, as well as hand washing when they returned home. Also, we ask about the use of gloves inside the house. Questions were asked about leaving the house and cleaning measures when returning, such as hand washing or bathing when returning home, taking off shoes and clothes to wash them.

#### Eating Habits

We evaluate the eating habits, to know the behavior of the participants during social isolation in some aspects such as diet, modifications and maintained habits. We use some elements based on the Spanish version of Eating Attitudes Test-26 (EAT-26) (18, 19), which is a validated instrument that assesses the risk of eating disorder.

#### Addictions

We include a section with some questions based and adapted from the 2016 National Addictions Survey (Encuesta Nacional de Adicciones 2016) (17, 20), to know the consumption of substances such as alcohol, tobacco and the consumption of illegal drugs prior to social isolation in the participants. Also, we asked if during the social isolation they started to consume tobacco, alcohol and illegal drugs.

#### Depression, Suicide, and Post-traumatic Stress Disorder

Finally, we included questions to screen for symptoms of depression and suicide behavior. For this study, we only wanted to know the presence or absence of depression in the participants. Thus, we apply items based on the Hospital Anxiety and Depression Scale (HADS) (21), which is a validated and reliable instrument that allows the detection of depression and anxiety in patients who attend a general medical clinic and has been recognized to measure the severity of these mood disorders. Also, we measure post-traumatic stress disorder due to the possibility of getting COVID-19. We use some questions based on the PTSD Symptom Scale-Self-Report (PSS-SR) (16), which is a validated instrument used to assess post-traumatic stress symptoms, where each item describes the symptom in terms of severity or frequency.

On the other hand, it is important to mention that the development of the questionnaire Mental Health in the Times of COVID-19 included a pilot version. This pilot study included 10 psychiatrists that answered the survey and gave their expert opinion; then, we adjusted the questions to avoid confusing language and terminology. These answers were not included in the analysis of results. To avoid risking the identity of the participants, no personal information was collected.

### Recruitment

The questionnaire was uploaded to the SurveyMonkey platform (22) and was distributed through social networks.

### Statistical Analysis

The survey was developed using the commercial tool SurveyMonkey and once the database was finished, it was downloaded in Excel format. The database was manually refined to eliminate questions without significant value. Once the debugging was completed, the base was loaded into the R environment for analysis (23). We evaluated variables using descriptive statistics. Quantitative variables we reported as means and standard deviations, while the categorical variables were represented in number of individuals and percentages (adjusted by the response rate of the section).

## Results

### General Description of the Respondents

A total of 1,011 surveys were collected; of those, 315 (31.16%) were men and 696 (68.84%) were women (Table 1). The mean age was 39.33 years with a S.D. ± 12.95. In relation to schooling, more than 40% (*n* = 483 individuals) had completed university studies and more than 20% (*n* = 278 individuals) had completed a postgraduate degree. More than 60% (*n* = 620) of participants were employees. In relation to marital status, almost half of the participants were single (48.47%, *n* = 490) and 41.64% (*n* = 421) were married. The mean body mass index reported by the participants was 29.01 with a S.D. ± 17.73; however, this could only be calculated in 1,001 individuals (99.01%), the rest of them did not respond the questions of weight and height. Of these 1,001 individuals who reported their BMI, 37.16% (*n* = 372) were overweight and 20.88% (*n* = 209) were obese.

**Table 1:** Sociodemographic characteristics of the respondents.

**Characteristics**	**Total (*n* = 1,011)**
Age	39.33 ± 12.95
**Gender**	
Male	315 (31.16)
Female	696 (68.84)
**Schooling level**	
Primary school	9 (0.89)
Mid-high school	65 (6.43)
High school	176 (17.41)
University	483 (47.77)
Postgraduate	278 (27.50)
**Occupation**	
Employed	620 (61.33)
Student	175 (17.31)
Housework	139 (13.75)
Unemployed	77 (7.62)
**Marital status**	
Single	490 (48.47)
Married	421 (41.64)
Divorced	77 (7.62)
Widower	23 (2.27)
Body mass index	29.01 ± 17.73
Normal	420 (41.96)
Overweight	372 (37.16)
Obesity	209 (20.88)
**Substance use**	
Alcohol	740 (77.98)
Tobacco	584 (60.64)
Illegal drugs	106 (11.10)

*The data are shown as mean ± standard deviation for quantitative variables and number of individuals and percentages for categorical variables*.

### Effect of Social Isolation During the COVID-19 Pandemic on Personal Hygiene Habits, Hand Washing, and Cleaning Measurements When Returning Home

In the personal hygiene habits section, we obtained a response rate of 93.37% (*n* = 944). Of these 944 individuals, 31.36% (*n* = 296) indicated that they washed their hands 2–4 times a day; 33.26% (*n* = 314) washed their hands 5–8 times a day; 35.17% (*n* = 332) wash their hands 8–16 times a day; finally, 0.21% (*n* = 2) reported that they did not wash their hands at all. Regarding any changes in their hand-washing habits, 17.27% (*n* = 163) did not make any changes, and 77.33% (*n* = 730) increased their hand-washing habits but without exaggeration. While 7.31% (*n* = 69) reported washing their hands so much that they have turned red, 0.74% (*n* = 7) wore gloves even inside their home. Those individuals who for any reason had to leave their home, upon returning 96.50% (*n* = 811) immediately washed their hands, 38.09% (*n* = 347) took off their shoes, 24.58% (*n* = 232) took off their clothes to wash them; 22.88% (*n* = 216) indicated that they had a shower immediately after returning home; while 3.91% (*n* = 37) reported that they continued their activities without making any changes. Finally, 8.69% (*n*=82) indicated that they had not left their home.

### Effect of Social Isolation During COVID-19 Pandemic on Eating Habits

The eating habits section presented a response rate of 95.54% (*n* = 966). Before social isolation 802 individuals (82.77%) reported normal eating habits; 6.81% (*n* = 87) considered that they were eating large amounts of food during the whole day, and 10.42% (*n* = 101) indicated that they had some compensatory behaviors (use of laxatives/more exercise, vomiting or fasting). Among individuals who reported to have compensatory behaviors, 8.98% (*n* = 87) were worried about gaining weight, so they tried to fast; 1.14% (*n* = 11) consumed laxatives or over-exercised and 0.31% (*n* = 3) reported having vomited after eating due to guilt.

During social isolation 372 individuals (38.51%) reported having modified their eating habits, where 18.32% (*n* = 177) reported looking much fatter than before social isolation. On the other hand, 13.35% (*n* = 129) reported having started a low-calorie diet, 5.80% (*n* = 56) have started consuming large amounts of food in short periods and had feelings of loss of control. In addition, 1.04% (*n* = 10) reported having started consuming laxatives, vomiting, or exercising excessively due to being concerned about gaining weight (Table 2).

**Table 2:** Modification of eating habits.

**Characteristics**	**Large amounts of food (*n* = 87)**	**Normal (*n* = 802)**	**Compensatory behaviors (*n* = 101)**	**Total (*n* = 966)**
Home laxatives/vomiting/exercise	1 (1.14)	5 (0.62)	4 (3.96)	10 (1.04)
Low calorie diet	17 (19.54)	96 (11.97)	16 (15.84)	129 (13.35)
Binge start	7 (8.05)	36 (4.49)	13 (12.87)	56 (5.80)
Distortion of perception	18 (20.69)	123 (15.34)	36 (35.64)	177 (18.32)
Maintained habits	22 (25.29)	540 (67.33)	32 (31.68)	594 (61.49)

*The data are shown as number of individuals and percentages*.

The greatest modification of eating habits occurred in people who already had compensatory behaviors before social isolation, as 68.32% (*n* = 69) of them increased their compensatory behaviors, while there were no significant differences among individuals who reported changes in their eating habits during social isolation. Some individuals reported consuming large amounts of food (49.43%, *n* = 13) while others reported having eating habits that were normal to their perception (48.15%, *n* = 260). The greatest increase was observed in individuals with previous compensatory behaviors and who are now much fatter than before social isolation (35.64%, *n* = 36).

### Effect of Social Isolation Due to the COVID-19 Pandemic on Tobacco, Alcohol, and Illegal Drugs Consumption

With regards to alcohol consumption there was a response rate of 93.57% (*n* = 946). Our survey indicates that 22.09% (*n* = 209) of participants reported never having consumed alcohol before social isolation, while 76.74% (*n* = 726) considered themselves alcohol users; of these, 81.13% (*n* = 589) reported consuming <5 glasses of alcoholic beverages per occasion, 18.87 % (*n* = 137) reported consuming more than 5 glasses of alcoholic beverages per occasion and 1.52% (*n* = 11) reported consuming alcoholic beverages to the point of losing consciousness and not remembering about it the following day.

During social isolation, 7.58% (*n* = 55) of participants reported daily alcohol consumption with or without an increasing their previous consumption, while 43.53% (*n* = 316) reported that they had not consumed alcohol during the pandemic. Interestingly, individuals who reported <5 glasses of alcoholic beverages per occasion before social isolation, 3.06% (*n* = 18) reported a daily consumption with an increase of glasses drank, while 3.06% (*n* = 18) indicated a daily consumption without increasing the number of glasses drank. Of those who consumed more than 5 glasses of alcoholic beverages per occasion before social isolation, the percentages of consumption were higher in 7.30% (*n* = 10) of those who consumed daily without increase, 5.11% (*n* = 7) showed an increase in their consumption. The highest percentage of increase was in individuals who drank alcoholic beverages to the point of losing consciousness before social isolation, where 18.18% (*n* = 2) of daily consumption was observed, with an increase in their level of consumption.

Regarding tobacco use, we obtained a response rate of 94.86% (*n* = 959). Of those, 60.79% (*n* = 583) had smoked at least once in their lives, of which 52.14% (*n* = 304) reported having smoked more than 100 cigarettes in their lifetime. Among those who had smoked at some point in their life, 1.20% (*n* = 7) started their tobacco use during social isolation, 3.26% (*n* = 19) had increased their consumption from 2 to 5 cigarettes and 1.03 % (*n* = 6) had increased their consumption by more than 5 cigarettes, while 11.66% (*n* = 68) had maintained their tobacco consumption in the same quantity.

In relation to the use of illegal drugs, we obtained a response rate of 90.21% (*n* = 912). Where 11.18% (*n*=102) reported having used an illegal drug in more than 5 occasions prior social isolation. Meanwhile, 0.44% (*n* = 4) started their consumption of some illegal drug during social isolation, with a consumption of more than 5 occasions during social isolation. In relation to previous users of illegal drugs, 9.80% (*n* = 10) reported an increase in consumption.

### Effect of Social Isolation During the COVID-19 Pandemic on Depression on Suicide Behavior

With regards to depression, we obtained a response rate of 97.63% (*n* = 987). We found that 35.16% (*n* = 347) of these individuals reported having symptoms of depression prior to social isolation. However, during social isolation, 46.10% (*n* = 455) reported having at least one symptom of depression, while 19.96% (*n* = 197) individuals reported at least one psychiatric symptom. Of those who had symptoms of depression before social isolation 9.02% (*n* = 89) reported the disappearance of symptoms of depression due to the effect of social isolation. While, 26.14% continued with the manifestation of depressive symptoms.

**Figure 1:**
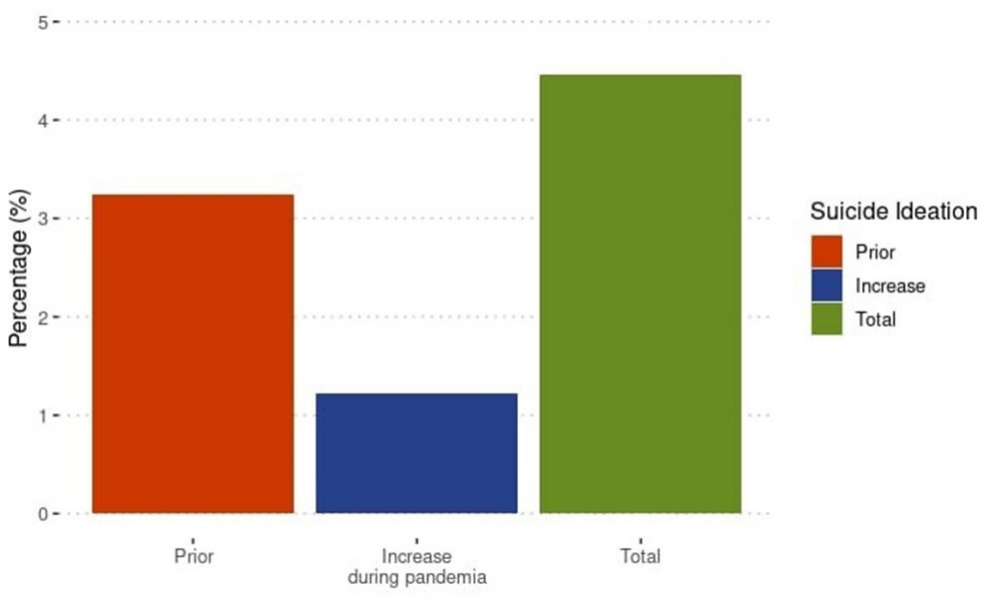
Results before and after of confinement.

Regarding suicidal ideation, 4.46% (*n* = 44) presented suicidal ideation due to social isolation, individuals with previous manifestations of depressive symptoms (12.40%, *n* = 32) had the highest presence of suicidal ideation, compared to those with onset of depressive symptoms during to social isolation (6.09%, *n* = 12) (Figure 1).

## Discussion

The World Health Organization has reported that when a disaster affects human lives, ~30–50% of the population suffer diverse psychological distress, anxiety and depression among other mood symptoms (24). Nowadays, the COVID-19 pandemic has had an impact on the lives of people worldwide. To reduce the spread of COVID-19 several countries around the world have set specific protocols in their nations that require an adequate disposition of their habitants to remain at home in isolation as much as possible; however the impact of COVID-19 on the populations' mental health is still unknown (22). Subsequently, we considered vital to evaluate the mental health of the general population in Mexico in order to help the general public in dealing with the current pandemic. For that reason, our study explored the possible effects of social isolation due to the COVID-19 pandemic on mental health in a sample of the Mexican population. In this study, we found an increase in symptoms of depression and suicide ideation and some people started the use of drugs and tobacco.

Studies that have explored changes in mental health due to COVID-19 have focused on depression and anxiety, where many authors have shown a concern about their increase, mainly in vulnerable populations such as older adults (25, 26). Our findings highlight warning symptoms in the Mexican population during this social isolation. Almost half (46.10%) of individuals surveyed reported having at least one symptom of depression. Other reports including the one by Tang et al., indicate that the prevalence of post-traumatic stress disorders and depression symptoms increased in a Chinese population due to social isolation (27). These outcomes reflect the urgency to address the psychological problems caused by social isolation to avoid a cyclical process of depression—social isolation (25). Additionally, it is probable that this type of psychological responses has affected the individuals' well-being and may persist long after the outbreak. Interestingly, we also found that some individuals who had depressive symptoms before social isolation, indicated that their depressive symptoms decreased, which could have been influenced by the activities that individuals carry out day by day. Some hypotheses talk about how individuals with depression can react in both negative and positive ways when faced to social distancing (28–31). Which opens a study area on the effects of social distancing and how it could influence individuals who already had depression.

We want to highlight that individuals with previous manifestations of depressive symptoms (12.40%, *n* = 32) also presented the highest presence of suicidal ideation when compared with those who started having symptoms of depression during social isolation; furthermore, 4.46% of them started with suicidal ideation during social distancing. As the increase in suicidal ideation could trigger suicide completion, the COVID-19 pandemic and social isolation could cause another public health problem.

Our results agree with previous reports; for instance, in a European population Fernández-Aranda et al., observed that individuals with eating disorders reported worsening in their symptomatology and additionally reported anxiety symptoms due to social isolation (32). Moreover, Zhou et al., observed that 20.9% of individuals with a preexisting psychiatric disorder worsened too (33). It is important to underline the fact that people with a prior psychiatric disease have higher risks during pandemics, for they are more susceptible to infections due to a cognitive impairment: they could have difficulties in accessing timely health services due to stigmatizations, lack of information, economic cost of mental health or just because it might be assumed that their symptoms will decrease in time (34). These individuals very often have emotional responses characterized by panic, anger and distress that could increase symptoms of depression, anxiety or suicide behaviors (35). During social isolation due to the COVID-19 pandemic, individuals have gone through changes in daily routines, there is fear of becoming infected, lack or miss-information regarding the route of transmission, stigmas or proximity to areas of high risk could lead them to manifest symptoms of depression or even suicide behavior (36). Therefore, the consequences of social isolation should be taken seriously.

An interesting finding in our study was that the majority of participants had university studies or higher, which indicates that social isolation in this population highly increases symptoms of depression and stress. This is opposite to what has been previously observed, as individuals with lower schooling levels are often associated with psychological distress when compared with individuals with high education (37), which opens the door for further studies to search for risk factors in Mexican with higher levels of education. Another important fact to consider is that psychological distress levels may exacerbate symptoms that were already present before social isolation (38, 39). Furthermore, it should be taken into consideration that the increased prevalence of self-reported physical symptoms is likely to have been caused by the psychological impact of the outbreak. In the view of continuing the social distancing, it is required the use of technology and social media to provide attention in real-time for this type of patients. Additionally, psychiatrics and health counselors should give consultations over the phone, as it can become an urgent necessity to receive psychiatric support and to prevent worsening consequences.

The main limitation of this work is the sample size, as we only included 1,011 surveys. Furthermore, a clear distribution is shown toward individuals with high schooling that could be due to their greater access to information technology and/or use of social networks; therefore, individuals with lower level of schooling can be under-represented. Also, the present work only screened a few symptoms of mental disorders and a deeper evaluation with validated diagnostic scales would be a perspective of the present work. Finally, we recognize that although the descriptive data described may show interesting results, we did not explore possible correlations and confounding factors, which could limit the interpretation of the findings. However, our study examines other mental health factors such as suicidal ideation so far not reported in the Mexican population during social isolation due to the first wave of COVID-19 (40).

## Conclusions

We identified that social isolation due to the COVID-19 pandemic has impacted the mental health of the Mexican population. Eating habits, depressive symptoms and suicide behavior showed an increase, except in some individuals where social isolation promoted a decrease of depressive symptoms. However, our findings should be judged considered the limitations above mentioned. Nonetheless, the present report presents the impact of pandemic in mental health in the Mexican population and could be considered for the construction of strategies for support and assistance in the Mexican population.

## Data Availability Statement

The datasets presented in this study can be found in online repositories. The names of the repository/repositories and accession number(s) can be found here: https://data.mendeley.com/datasets/znzbryfw53/1.

## Ethics Statement

The studies involving human participants were reviewed and approved by the Research Ethics Committee of the Juárez Autonomous University of Tabasco (103/CIP-DACS/2020) in Mexico. The patients/participants provided their written informed consent to participate in this study.

## Author Contributions

ADG-M, JJM-M, TBG-C, IEJ-R, HN, and CAT-Z: study concepts, design, data acquisition, and data analysis. ADG-M, JJM-M, MLL-N, TBG-C, IEJ-R, HN, CAT-Z, and RGC-A: interpretation, manuscript preparation, manuscript editing, and manuscript review. ADG-M, JJM-M, TBG-C, HN, and CAT-Z: statistical analysis. All authors contributed to the article and approved the submitted version.

## Conflict of Interest

The authors declare that the research was conducted in the absence of any commercial or financial relationships that could be construed as a potential conflict of interest.

## Publisher's Note

All claims expressed in this article are solely those of the authors and do not necessarily represent those of their affiliated organizations, or those of the publisher, the editors and the reviewers. Any product that may be evaluated in this article, or claim that may be made by its manufacturer, is not guaranteed or endorsed by the publisher.
